# Atherosclerosis and Cardiovascular Complications in People Living with HIV: A Focused Review

**DOI:** 10.3390/idr16050066

**Published:** 2024-09-01

**Authors:** Michele Salvatore Paternò Raddusa, Andrea Marino, Benedetto Maurizio Celesia, Serena Spampinato, Carmen Giarratana, Emmanuele Venanzi Rullo, Bruno Cacopardo, Giuseppe Nunnari

**Affiliations:** 1 Department of Clinical and Experimental Medicine, University of Messina, 98124 Messina, Italy; michelepat93@gmail.com (M.S.P.R.); serenaspampinato93@gmail.com (S.S.); carmengiarratana15@gmail.com (C.G.); emmanuele.venanzirullo@unime.it (E.V.R.); 2 Department of Clinical and Experimental Medicine, Unit of Infectious Diseases, ARNAS Garibaldi Hospital, University of Catania, 95123 Catania, Italy; bmcelesia@gmail.com (B.M.C.); cacopard@unict.it (B.C.); giuseppe.nunnari1@unict.it (G.N.)

**Keywords:** HIV, HIV and cardiovascular diseases, AIDS, HIV comorbidities, non-AIDS-related comorbidities

## Abstract

The intersection of Human Immunodeficiency Virus (HIV) infection and cardiovascular disease (CVD) represents a significant area of concern; advancements in antiretroviral therapy (ART) have notably extended the life expectancy of people living with HIV (PLWH), concurrently elevating the prevalence of chronic conditions such as CVD. This paper explores the multifaceted relationship between HIV infection, ART, and cardiovascular health, focusing on the mechanisms by which HIV and ART contribute to increased cardiovascular risk, including the promotion of endothelial dysfunction, inflammation, immune activation, and metabolic disturbances. We highlight the critical roles of HIV-associated proteins—Tat, Nef, and gp120—in accelerating atherosclerosis through direct and indirect pathways that exacerbate endothelial damage and inflammation. Additionally, we address the persistent challenge of chronic inflammation and immune activation in PLWH, factors that are strongly predictive of non-AIDS-related diseases, including CVD, even in the context of effective viral suppression. The impact of ART on cardiovascular risk is examined, with particular attention to the metabolic implications of specific ART regimens, which can influence lipid profiles and body composition, thereby modifying CVD risk. The therapeutic potential of statins, aspirin, and emerging treatments such as PCSK9 inhibitors in mitigating cardiovascular morbidity and mortality among PLWH is discussed, alongside considerations for their use in conjunction with ART. Our review underscores the necessity for a comprehensive, multidisciplinary approach to cardiovascular care in PLWH, which integrates vigilant cardiovascular risk assessment and management with HIV treatment. As we navigate the evolving landscape of HIV care, the goal remains to optimize treatment outcomes while minimizing cardiovascular risk, ensuring that the gains in longevity afforded by ART translate into improved overall health and quality of life for PLWH.

## 1. Introduction

In 2022, an estimated 39 million people worldwide (ranging from 33.1 million to 45.7 million) were living with HIV. Among them, 86% (varying from 73% to over 98%) were aware of their HIV status, 76% (between 65% and 89%) were receiving antiretroviral therapy (ART), and 71% (from 60% to 83%) had achieved viral suppression [1].

Due to the effectiveness of ART, the mortality rate among people living with HIV (PLWH) has decreased, leading to a significant increase in their life expectancy by more than 50 years [2,3,4,5,6]. At the same time, a global rise in chronic diseases such as cardiovascular disease (CVD) has been observed within this population [7]. PLWH face higher rates of CVD risk factors compared to the general population, including dyslipidemia, diabetes, metabolic syndrome, smoking, hypertension, and drug use. Additionally, HIV-specific factors like ART, chronic inflammation, and immune activation also contribute to the increased risk of CVD [8]. Hypertension affects 21.2% of HIV-positive individuals versus 15.9% of HIV-negative individuals, while dyslipidemia is present in 23.3% of HIV-positive compared to 17.6% of HIV-negative patients [9].

A meta-analysis and systematic review found that HIV-positive individuals have a 1.5 to 2 times higher risk of MI compared to their HIV-negative counterparts [10,11]. A study reported MI rates of 11.13 per 1000 person-years in HIV-positive patients versus 6.98 in HIV-negative patients, even after adjusting for confounding factors like age, sex, and traditional cardiovascular risk factors [12].

Atherosclerosis, a chronic inflammatory condition of the arterial wall, is the major precursor of CVD, leading to severe outcomes. Advanced stages of atherosclerosis are characterized by damage to the intimal-media layer of the arterial wall and the buildup of plaques. These plaques can erode or rupture, leading to thrombotic events with potentially fatal consequences, like myocardial infarction and stroke. The development of atherosclerosis involves a complex interplay of factors, from lipid accumulation to chronic inflammation within the arterial wall [13]. As shown by several studies involving coronary CT angiography, PLWH are more likely either to have coronary artery plaque than those without HIV or to have a greater extent of non-calcified plaque, which is particularly prone to rupture [14,15]. All this evidence highlights a critical need for ongoing monitoring and tailored interventions to manage cardiovascular health in HIV-positive populations.

## 2. HIV-Mediated Molecular Mechanisms

Peripheral artery disease (PAD) is notably more prevalent among PLWH than in the general population, serving as a common clinical manifestation of atherosclerosis and signaling an increased risk of cardiovascular events such as myocardial infarction (MI) and stroke. Its prevalence in PLWH is 20.7%, compared to just 1% in those not infected [16,17]. InResearch suggests that HIV-associated proteins, including Tat, Nef, and gp120, play significant roles in the onset and acceleration of atherosclerosis. These proteins induce endothelial dysfunction through various pathways.

Gp120 is part of the envelope protein complex of HIV. It binds to the CD4 receptor on the surface of host T cells and other immune cells, being the first step in the fusion process of the viral membrane with the host cell membrane, leading to viral entry into the host cell.

Specifically, HIV gp120 protein disrupts normal endothelial cell function, damaging proteins that maintain cell junctions, increasing blood vessel permeability, and triggering a chain of pathological changes in vascular tissue. These changes can advance stromal degeneration, necrosis, sclerosis, and hyalinosis, potentially leading to various complications, including cardiovascular issues [18,19].

Gp120’s toxic effects on endothelial cells are mediated by activation of caspase-3, involved in apoptosis; activation of Bax, regulating apoptosis; involvement of protein kinase C (PKC) in cellular signaling; activation of mitogen-activated protein kinase p38 (MAPK), affecting cellular processes; and direct interaction with the CXCR4 co-receptor, affecting immune response and cell adhesion [18].

Moreover, gp120 activates the lectin-like oxidized low-density lipoprotein receptor-1 (LOX-1), leading to reactive oxygen species (ROS) production, nuclear factor-kappa B (NF-kB) activation, and increased levels of pro-inflammatory factors, contributing to vascular remodeling and enhancing plaque vulnerability [20,21].

Tat protein promotes viral transcription by enhancing the elongation of RNA transcripts from the HIV long terminal repeat (LTR), which is critical for the production of new viral particles.

Tat protein inducts the release of proinflammatory cytokines, concurrently prompting the endothelial expression of IL-1b, MCP-1, vascular cell adhesion protein 1 (VCAM-1), and E-selectin through the NF-kB-dependent pathway [22]. Furthermore, Tat induces oxidative stress and a mild inflammatory response in the endothelium by activating NADPH oxidase [22]. Tat protein stimulates production of IFN-γ, TNF-α, IL-6, and IL-17 and the apoptosis of endothelial cells; this causes atherosclerotic lesions due to an easier accumulation of low-density lipoprotein due to sub-endothelial space enhanced permeability [23].

The Nef protein is involved in downregulating the expression of certain cell surface receptors (such as CD4 and MHC I) on infected cells, helping HIV to evade the host immune system.

Nef protein amplifies the adherence of T-lymphocytes to endothelial cells, impeding their diapedesis and migration into the subendothelial space, potentially in association with the activity of matrix metalloproteinases (MMPs) [19,24]. Nef’s role extends to the alteration of cholesterol homeostasis in endothelial cells through the phosphorylation of caveolin-1, disrupting high-density lipoprotein (HDL)-mediated cholesterol efflux and promoting the formation of foam cells [24].

**Figure 1:**
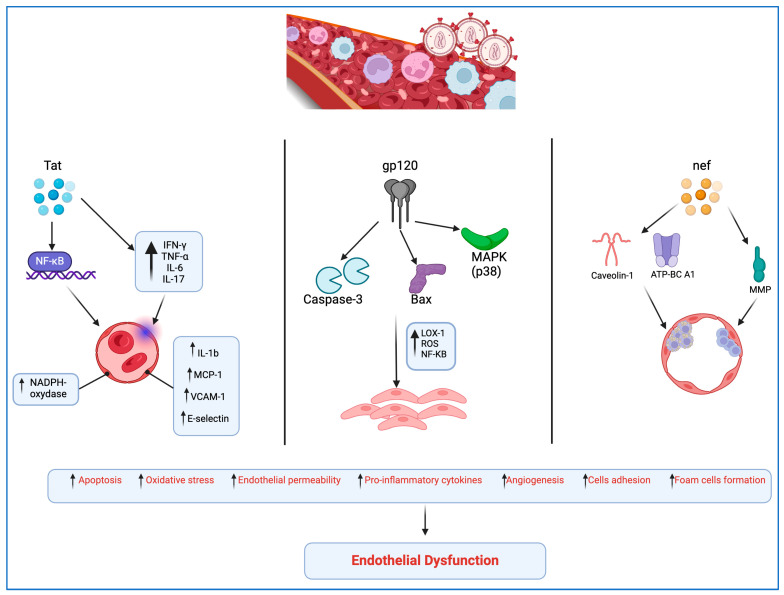
HIV-mediated molecular mechanisms leading to endothelial dysfunction (created with BioRender.com).

Additionally, Nef interferes with the normal function of the ATP-binding cassette transporter A1 (ATP-BC A1), hindering cholesterol efflux from infected macrophages and fostering foam cell development [25] (Figure 1).

Moreover, HIV ribonucleic acids bind to Toll-like receptor 8 (TLR-8), facilitating the transformation of monocytes/macrophages into foam cells within the vascular wall in a dose-dependent manner. This mechanism further highlights the complex interaction between HIV infection and cardiovascular disease, emphasizing the multifaceted impact of HIV on vascular health and disease progression in PLWH [26].

## 3. Role of Chronic Inflammation and Immune Activation

### 3.1. Inflammatory Markers and Immune Activation

PLWH are characterized by noticeable abnormal inflammation and immune activation, which are considered strong predictive indicators of mortality and non-AIDS diseases developing, such as CVD [27,28,29,30,31].

Chronic inflammation in PLWH is marked by elevated levels of pro-inflammatory cytokines, including IL-6, IL-1β, TNF-α, VCAM-1, ICAM-1, CRP, and D-dimer [32]. These inflammatory markers remain elevated compared to the general population even after achieving viral suppression through ART [33]. This persistent inflammation contributes significantly to the increased cardiovascular risk in PLWH.

### 3.2. Demographic Influences on Inflammation and CVD

Additionally, demographic factors such as age, gender, and race significantly influence the progression of cardiovascular diseases in this population. Studies have shown that older age is associated with higher levels of inflammation and a greater burden of atherosclerosis in PLWH [34]. Similarly, men with HIV are at a higher risk of developing cardiovascular diseases compared to women, partly due to differences in inflammation and immune activation [35]. Racial disparities also play a crucial role; for example, African Americans living with HIV tend to have higher levels of inflammatory markers and a greater prevalence of cardiovascular diseases compared to their Caucasian counterparts [36].

The interplay between chronic inflammation, immune activation, and these demographic factors necessitates a comprehensive approach to understanding and managing cardiovascular risks in PLWH. Addressing these risks requires not only effective viral suppression but also targeted interventions to reduce inflammation and improve cardiovascular health outcomes. Strategies such as the use of anti-inflammatory medications and lifestyle modifications, including diet and exercise, may be beneficial in this regard [37].

### 3.3. Gut Microbiota and Systemic Inflammation

Gut microbiota dysbiosis and increased gut permeability are significant contributors to systemic inflammation in PLWH, driven by HIV’s disruption of gut homeostasis through targeting mucosal CD4+ T cells. This disruption leads to epithelial cell death, diminished cell–cell adhesion, and microbial translocation, where products like lipopolysaccharide (LPS) enter the bloodstream, exacerbating immune activation and chronic inflammation [37,38,39]. This population exhibits elevated levels of inflammation markers, including IL-6, IL-1β, TNF-α, VCAM-1, ICAM-1, CRP, and D-dimer, many of which remain elevated even after viral suppression [23,32,33]. The imbalance in the gut microbiota is closely linked to the onset and progression of various diseases, including cardiovascular diseases and immune disorders, through alterations in gut permeability and subsequent movement of pathogens into the bloodstream [40,41]. These alterations activate pathways like TLR4, further increasing cytokine production and pro-inflammatory factors [38,42,43]. Restoring gut microbiota balance with probiotics or prebiotics may help manage this chronic inflammation in PLWH.

### 3.4. Role of Activated T Cells and Monocytes

Activated T cells and monocytes play a role in sustaining inflammation, hypercoagulation, and endothelial dysfunction, all pivotal in the progression of cardiovascular disease [24,44]. Monocytes maintain the HIV reservoir among individuals who have achieved virological suppression, contributing to persistent immune activation and inflammation. The activation of monocytes persists even following the initiation of treatment, with a noted correlation between the thickness of the carotid intima-media and the expression of surface markers/receptors CD11b and C-X3-C motif receptor-1 (CX3CR1) on activated monocytes [43].

Elevated levels of soluble CD163 (sCD163), an endocytic receptor for haptoglobin-hemoglobin complexes, have been associated with non-calcified coronary plaques and arterial inflammation in individuals living with HIV [45]. IL-6 stimulates the conversion of monocytes into activated macrophages via the macrophage colony-stimulating factor (M-CSF). Foam cells, which are heterogeneous macrophages filled with cholesterol, form the basic building blocks of arterial lesions caused by atherosclerosis. Their accumulation results in fatty patches that eventually become plaques, playing a direct role in the development of atheroma [46].

Lipid accumulation in dendritic or macrophage cells triggers the activation of the inflammasome, including NLRP3, which is crucial in the pathophysiology of HIV-induced inflammation and plays a role in the initiation and progression of atherosclerosis [47,48].

### 3.5. MAP-Kinase Signaling and HIV-Related Inflammation

The replication of HIV-1 can be favorably regulated by the MAP-kinase signal loop, with ERKs, p38 MAP-kinases, and cJun NH2-terminal kinases (JNK) linked to HIV-related inflammation and the promotion of atherosclerosis through various mechanisms, including the activation of NF-kB and the promotion of ICAM-1 expression, VEGF-induced endothelial cell permeability, and the development of reactive myocardial fibrosis [49,50,51,52].

MAP-kinase and NF-kB disruptions, therefore, have a dual function in HIV-induced inflammation and vascular lesions associated with atherosclerosis.

### 3.6. Perivascular Adipose Tissue and HIV-Related Atherosclerosis

The function of perivascular adipose tissue (PVAT) in the context of low-grade inflammation and the development of CVD among PLWH is significant. Proinflammatory adipokines and cytokines produced by PVAT’s defective phenotype diffuse through the vascular wall or the vasa vasorum [53]. Remarkably, the release of anti-inflammatory cytokines, particularly IL-6 and IL-10, is also a hallmark of HIV-associated inflammation. This results in a destabilized PVAT phenotype due to the overproduction of hematogenous CD163+ macrophages and insulin resistance [54]. CD163+ macrophages are associated with increased angiogenesis, microvascular permeability, and endothelial inflammation of the plaque wall. They also lead to the loss of VE-cadherin by overproduction of VEGF-A, altering the morphology and integrity of endothelial cells [55]. Morphologic variations exist between “ordinary” atherosclerosis and HIV-related atherosclerosis. Intravascular ultrasound showed that those living with HIV had greater rates of hypoechoic plaques and lower plaque burdens [56]. In a multicenter study conducted by Wendy S. et al. using computed tomographic angiography on 1001 individuals (618 HIV-infected and 383 uninfected men who have sex with men), it emerged that noncalcified plaque is more common and prominent in PLWH than in the control group [14]. Increased carotid IMT has been observed in the setting of HIV in numerous investigations. This has been linked with higher mortality and inflammatory marker levels [34,57]. In a cross-sectional study of 81 participants, it was shown that people living with HIV have elevated levels of arterial inflammation than group control, as determined by fluorodeoxyglucose-positron emission tomography/computed tomography; this is associated with macrophage activity producers [58].

## 4. Metabolic Impairment in PLWH

Individuals with untreated HIV infection exhibit a higher prevalence of cardiovascular abnormalities compared to the general population, with endothelial dysfunction and carotid intima-media thickening being especially prominent. Dyslipidemia, insulin resistance, and changes in body composition are highly prevalent among PLWH and are significant contributors to the elevated risk of CVD observed in this population [59].

The study by Masenga et al. [60] emphasizes that the prevalence of hypertension and metabolic syndrome is higher in HIV-positive individuals, partly due to impaired glucose metabolism and increased insulin resistance. These findings are supported by Aurpibul et al. [61], who report that perinatally infected youth on ART also exhibit significant metabolic syndrome markers, indicating that metabolic complications begin early in the HIV-positive population.

In the absence of ART, HIV infection leads to significant reductions in total cholesterol (TC), high-density lipoprotein cholesterol (HDL-C), and low-density lipoprotein cholesterol (LDL-C). These lipid abnormalities become more pronounced as HIV progresses, with individuals at advanced stages, such as AIDS, exhibiting the most severe metabolic impairments [23,45,59,62]. Specifically, those with AIDS have notably higher levels of triglycerides compared to individuals with HIV but without AIDS and the general population. These patterns of lipid dysregulation mirror those observed in other states of chronic infection and inflammation. Consistent findings across different studies indicate that these lipid abnormalities contribute to the heightened cardiovascular risk seen at various stages of HIV infection [45].

PLWH have higher concentrations of leptin and lower levels of adiponectin; these imbalances can lead to increased central fat mass, worsened insulin sensitivity, higher glucose concentrations, and an elevated risk of cardiovascular disease [44].

HIV infection frequently results in changes to body composition. Patients who were first diagnosed during the ART era often experienced an increase in visceral fat around the abdomen and a reduction in subcutaneous fat. The changes in fat distribution have been varied and associated with the accumulation of ectopic adipose tissue in the muscle and liver, as well as with insulin resistance. These alterations are attributable to several factors, including the effects of ART itself. The use of specific thymidine NRTIs has been linked to the deposition of ectopic adipose tissue in the muscle and liver, loss of subcutaneous fat, and vascular inflammation [63].

With the initiation of ART, elevations in both visceral and subcutaneous fat are commonly observed due to more effective treatment options, and rates of generalized obesity are increasing among PLWH, regardless of region [62]. Changes in body composition, including the accumulation of extra-visceral adipose tissue, have been associated with overall mortality. Furthermore, these changes have been linked to an increase in the quantity of both calcified and noncalcified coronary plaques, highlighting the complex interplay between HIV infection, ART, and cardiovascular risk [64].

## 5. Role of Co-Infections

Co-infections significantly influence the development and exacerbation of atherosclerosis in HIV-infected individuals by amplifying the risk factors associated with cardiovascular diseases. The persistent inflammation observed in HIV-infected patients is further sustained by co-infections with other viruses, such as hepatitis C virus (HCV), hepatitis B virus (HBV), cytomegalovirus (CMV), and Epstein–Barr virus (EBV). Studies, including those by Márquez et al., indicate that HIV/HCV co-infected patients exhibit higher levels of inflammatory markers such as sCD14 and IL-6 compared to those with chronic hepatitis or HIV mono-infection. Elevated levels of pro-inflammatory and profibrogenic chemokines such as MCP-1 and eotaxin have been linked to HIV/HCV co-infection, suggesting a heightened proinflammatory response. This is compounded by the increased risk of metabolic disorders in HIV/HCV co-infected individuals, further elevating their risk for cardiovascular diseases due to enhanced endothelial dysfunction and chronic inflammation. The evidence underscores the need for comprehensive management strategies that address not only HIV but also co-infections to mitigate the increased risk of cardiovascular diseases, including atherosclerosis, in this population [65,66].

Furthermore, co-infections like CMV can damage epithelial connections, leading to bacterial translocation and persistent gastrointestinal inflammation, which can draw HIV-target cells to the site, promoting HIV persistence. CMV also enhances HIV persistence by promoting the survival of HIV-infected cells, stimulating inhibitory immune pathways, and directly transactivating latent HIV. The association of higher levels of sCD14, CXCL10, and genetically intact HIV in individuals co-infected with HIV and HBV underlines the impact of immune activation on the persistence and complexity of HIV infection [67,68,69,70].

## 6. Role of ART

### 6.1. Impact on Life Expectancy and Cardiovascular Risks

The life expectancy of PLWH has significantly increased because of the efficiency of ART, which has also reduced the death rate among PLWH [71].

The literature suggests that certain ART regimens may play a role in stimulating endothelial dysfunction and promoting atherosclerosis. HIV, the immune reconstitution response, and antiretroviral therapy may provoke early endothelial activation, potentially making them pro-atherogenic factors [72,73].

Conversely, antiretroviral therapy is beneficial in mitigating the proinflammatory impact of HIV infection. Post-treatment, PLWH exhibits a significant decline in the concentrations of IL-6, IL-1β, D-dimer, ICAM-1, VCAM-1, and TNF-α during ART.

### 6.2. Insights from Clinical Studies: SMART and START

The “Strategies for Management of Antiretroviral Therapy” (SMART) and the “Strategic Timing of Antiretroviral Treatment” (START) studies have provided valuable insights into antiretroviral therapy and its optimal timing in relation to HIV infection and CVD. The SMART study showed that continuous ART use in individuals with a CD4+ cell count below 350/µL resulted in a decrease in AIDS-related complications and occurrences of CVD. Those who delayed or interrupted treatment faced a higher risk of cardiovascular disease. The START study found that immediate ART administration led to a 40% reduction in AIDS-related cases, although it did not significantly impact the occurrence of CVD [74].

Both the SMART and START studies support the notion that antiretroviral therapy should decrease the frequency of CVD, but ART alone may not suffice as a preventive measure against cardiovascular events in individuals with HIV.

### 6.3. Dyslipidemia and ART Regimens

Dyslipidemia is a primary risk factor for cardiovascular disease and atherosclerosis. Byonanebye et al.’s study from the International Cohort Consortium of Infectious Disease (RESPOND) in 2021 found that patients, both ART-naïve and ART-experienced, treated with Integrase Strand Transfer Inhibitor (INSTI)-based therapy were less likely to experience dyslipidemia compared to those treated with protease inhibitor (PI)-based therapy [75].

PIs have been associated with an increase in hypertriglyceridemia and levels of total and LDL cholesterol [76]; ritonavir, in particular, has been linked to hyperlipidemia and demonstrated to increase cardiovascular mortality, risk of myocardial infarction, and have a minor but statistically significant impact on the progression of carotid-wall thickness [77,78].

Furthermore, ritonavir-boosted darunavir use was associated with an increased risk of CVD [79].

Reductions in adiposity and leptin secretion lead to decreased endothelial leptin signaling, subsequently reducing NO bioavailability mediated by CCR5 and NADPH oxidase 1, directly causing ritonavir-associated endothelial dysfunction [78].

Although newer PIs have less severe metabolic side effects, PLWH with dyslipidemia should be treated with consideration for the impact of these drugs on lipids [51]. Moreover, a connection between PI treatment and increased intima–media thickness (IMT) comparing Doppler data has been shown [80].

First-line HIV treatment guidelines no longer include boosted atazanavir and darunavir; however, darunavir is used as salvage therapy, part of two-drug regimens, or as alternatives for first-, second-, and third-line regimens. Finally, considering the baseline risk for cardiovascular disease is advisable before using a PI-based regimen, especially in the elderly PLWH [81,82].

Currently, INSTIs are the cornerstone of antiretroviral therapy. INSTIs are superior to non-nucleoside reverse transcriptase inhibitors (NNRTIs) and PIs regarding virological efficacy, tolerability, and the risk of drug–drug interactions [83,84].

Weight gain has been linked to INSTI-based ART in both treatment-naïve and treatment-experienced individuals [85,86]; those newly starting INSTI-based ART have reported an elevated incidence of arterial hypertension and diabetes [87,88].

### 6.4. Considerations for Switching ART Regimens

The “Surveillance Cohort Long-Term Toxicity Antiretrovirals” (SCOLTA), an observational cohort trial involving PLWH treated with efavirenz or a ritonavir-boosted PI-based regimen, switching to dolutegravir, elvitegravir, or rilpivirine, observed a decrease in LDL-C among patients who transitioned from a ritonavir-boosted PI to elvitegravir. Additionally, a reduction in total cholesterol was noted across all groups except for the efavirenz to elvitegravir switch group upon transitioning to an INSTI-based regimen. However, no appreciable change in triglycerides was observed among those who switched to an INSTI-based regimen, emphasizing the nuanced impacts of different ART regimens on lipid profiles [89].

According to recent antiretroviral treatment guidelines, regimens based on tenofovir, a nucleoside reverse transcriptase inhibitor (NRTI), are among the first-line therapies recommended for most PLWH. Treatments based on tenofovir disoproxil fumarate (TDF) have been associated with higher plasma levels of tenofovir, with potential adverse effects on kidney and bone. Regimens based on tenofovir alafenamide (TAF) result in fewer negative effects and achieve higher intracellular levels of tenofovir-diphosphate than TDF, albeit with lower plasma levels of tenofovir. On the contrary, TDF exhibits a “statin-like” effect due to indirect mechanisms on lipid metabolism, adipose tissue, and liver function, whereas TAF showed a “neutral” effect on lipid assessment. According to that, switching from TDF to TAF could result in higher levels of total cholesterol due to the end of the TDF-statin effect [90,91]. In addition, TAF, compared to TDF, is associated with greater weight gain, both after the switch and in treatment-naïve patients [92,93]. According to Gregory D. Huhn et al., lipid changes with TAF as part of co-formulated regimens do not significantly alter CVD risk profiles compared to TDF [94].

NNRTIs have varying effects on lipids. Etravirine had a neutral effect on TC, LDL-C, and TG. Compared to nevirapine and rilpivirine, efavirenz raises TG and LDL-C more than the others [95].

Switching from a nevirapine-based ART to a rilpivirine-based ART resulted in a mean decrease in LDL-C and total cholesterol [96]. Doravirine (DOR) appears to be well tolerated in both naïve and experienced PLWH. In the phase 3 DRIVE-FORWARD trial, after 48 weeks of treatment, a better lipid profile was observed in adults naive treated with two NRTIs and DOR than darunavir/ritonavir [97]. In comparison to efavirenz/emtricitabine/TDF, DOR in combination with lamivudine and tenofovir disoproxil fumarate showed minimal changes in LDL-C and non-HDL-C at week 48 in the phase 3 DRIVE-AHEAD trial [98].

In individuals with HIV suppression lasting more than six months and no history of virologic failure, the phase 3 DRIVE-SHIFT trial showed, twenty-four weeks after the switch from a stable ART to once-daily DOR/3TC/TDF, a reduction in fasting lipids that persisted until week 144 [99]. Maggi et al., in a real-life setting involving 295 subjects on RPV and 256 on DOR-based regimens, found that both regimens improved the lipid profile [100]. Doravirine was found to be more effective than rilpivirine in lowering the ratio of total cholesterol to high-density lipoprotein cholesterol. These findings suggest that doravirine may offer more benefits than rilpivirine for managing lipid levels and transaminases.

**Figure 2:**
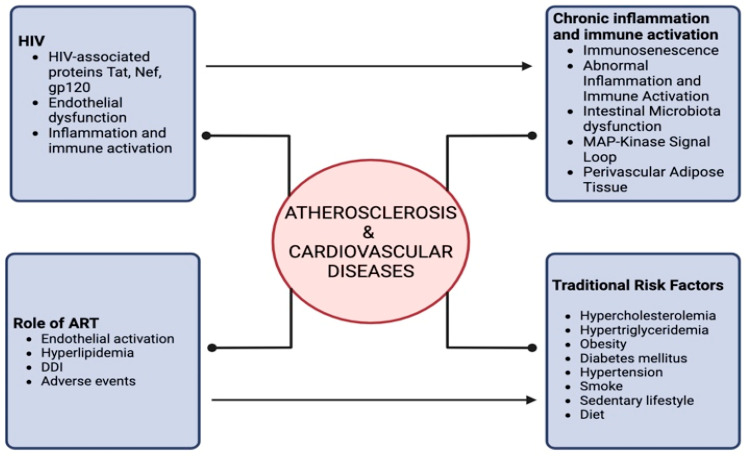
Correlation between HIV and atherosclerosis/cardiovascular diseases.

Figure 2 summarizes the interplay between HIV infection and atherosclerosis.

## 7. Treatment and Prevention

According to international guidelines, statins and aspirin play a critical role in reducing cardiovascular-related morbidity and mortality in PLWH, just as they do in the non-infected population (Table 1).

In a multicenter, prospective cohort study, including 1182 HIV patients, conducted to investigate the use of statins and acetylsalicylic acid (ASA) in PLWH in clinical practice, it emerged that the prescription of statins and ASA in HIV-infected patients is largely suboptimal, as only about 50% of patients requiring statins and ASA are properly treated [101].

The role of aspirin as preventive therapy is contentious. The ASPREE study found that using low-dose aspirin as the primary preventive measure for elderly subjects without HIV led to a significantly increased risk of serious bleeding but did not significantly reduce the risk of cardiovascular disease over a 5-year period when compared to a placebo [102].

The American College of Cardiology (ACC) [103] and the American Heart Association (AHA) Guidelines [104,105] on the primary prevention of cardiovascular disease recommend aspirin primarily for the secondary prevention of atherosclerotic cardiovascular disease. However, current evidence suggests that aspirin may not be beneficial for routine cardiovascular disease prevention [103].

It also indicates that adults between the ages of 50 and 59 who have a 10-year CVD risk of more than 10%, who are willing to take low-dose aspirin for at least ten years, and are not at an increased risk for bleeding, can be considered for aspirin treatment (Grade B recommendation). Individuals between the ages of 60 and 69 with a 10-year CVD risk of over 10% should consider the benefits and risks of long-term low-dose aspirin. Those most likely to benefit are without a history of bleeding, have a life expectancy of at least ten years, and are willing to use low-dose aspirin for that duration (Grade C recommendation).

Aspirin, the most widely used antiplatelet medication, may exhibit decreased therapeutic efficacy in PLWH [106].

Studies have shown that aspirin had no effect on endothelial dysfunction or immune activation indicators in a randomized clinical trial with PLWH on ART [107].

In another trial examining the effects of clopidogrel and aspirin, aspirin did not reduce platelet-induced endothelial inflammation or platelet activation [108].

The latest European AIDS Clinical Society (EACS) guidelines [109] state that, regardless of cholesterol levels, anyone with a history of vascular disease or those at high enough risk of CVD that cannot meet LDL-C goals should take statins. If a patient is at high risk due to statin intolerance, drug–drug interactions between ART and high-intensity statins, or the inability to achieve LDL-C goals while on ezetimibe and/or statins, the use of PCSK9 inhibitors and/or bempedoic acid should be considered. For very high-risk individuals (such as diabetics and post-myocardial infarction patients with another CV risk factor and triglycerides > 150 mg/dL or >1.7 mmol/L), icosapent ethyl (EPA) should be considered as an adjunct to statin therapy.

Simvastatin is contraindicated in individuals receiving treatment with protease inhibitors/ritonavir; instead, atorvastatin and rosuvastatin should be started at a low dose. This recommendation also applies to patients receiving non-nucleoside treatment. Simvastatin and lovastatin should be avoided in PLWH since they are extensively metabolized by the CYP system and can have their levels elevated by more than 500% when coadministered with CYP inhibitors [110]. Pravastatin dosages should be lowered when combined with darunavir/ritonavir.

Rosuvastatin and atorvastatin, the two highest-intensity statins, have mild interactions with ART. At their maximum commonly prescribed doses, these statins can reduce LDL-C by more than 50% [95,110]. In a randomized controlled trial, rosuvastatin 10 mg compared to placebo reduced vascular inflammation, monocyte activation indicators, and several other inflammatory markers in PLWH [111].

Pitavastatin is preferred over other statins because it has fewer negative effects on glucose metabolism, results in a higher increase in HDL-C, and has fewer drug–drug interactions [112].

Numerous studies have linked statin medication to a beneficial reduction in the common carotid artery intima–media thickness in both the general population and PLWH, encouraging the use of high-intensity statin in patients with subclinical atherosclerosis regardless of LDL-C levels or calculated 10-year risk [113].

In the REPRIEVE trial, Steven K. Grinspoon et al. randomized 7769 HIV-positive individuals taking antiretroviral medication and having a low-to-moderate risk of cardiovascular disease to receive either a placebo or daily pitavastatin calcium (at a dose of 4 mg) [114].

Participants using pitavastatin had a lower risk of major adverse cardiovascular events compared to those receiving a placebo, with a 35% reduction in risk over a median follow-up period of 5.1 years, leading to the early termination of the trial.

If a statin is not well tolerated or if the treatment target (a reduction in the percentage of LDL-C or a level of LDL-C greater than 70 mg/dL) has not been met, ezetimibe may be considered for PLWH who are at a higher risk of CVD. Saeedi et al., in a trial involving 43 PLWH, reported that after 12 weeks of treatment with either rosuvastatin 10 mg daily with ezetimibe 10 mg daily or rosuvastatin 20 mg daily alone, participants in both treatment groups showed comparable levels of LDL-C reduction. While there was no discernible change in HDL-C, the ezetimibe add-on arm showed a significant drop in TG from baseline. Finally, both treatments were well tolerated regarding side effects [115].

Given the interactions between certain ARTs and statins, PCSK9 inhibitors have emerged as a promising alternative strategy for reducing LDL-C concentrations. In a double-blind trial, Boccara et al. evaluated the safety and efficacy of evolocumab over 24 weeks in PLWH who had mixed dyslipidemia or hypercholesterolemia and were on maximally tolerated statin ttherapy [116]. Evolocumab treatment led to a reduction in LDL-C by more than 56% compared to placebo, while the placebo-corrected percentages of patients achieving an LDL-C level of <70 mg/dL and an LDL-C reduction of ≥50% were 65.4% and 71.9%, respectively. Moreover, evolocumab treatment resulted in an increase in HDL-C and a decrease in triglycerides and atherogenic lipid markers (non-HDL-C, ApoB, total cholesterol, VLDL-C, and Lp[a]). Finally, in people living with HIV, evolocumab was safe and well-tolerated.

**Table 1:** Treatment and prevention summary and recommendation.

Recommendation	Population	Details	Study/Source	Efficacy
Aspirin (for prevention)	Elderly without HIV	Increased risk of serious bleeding, no significant reduction in cardiovascular disease	ASPREE study [102]	Not recommended for primary prevention
Aspirin	Adults aged 50–59 with >10% 10-year CVD risk	Considered if willing to take for at least ten years and not at increased bleeding risk	ACC [103] and AHA Guidelines [104,105]	Grade B recommendation
	Adults aged 60–69 with >10% 10-year CVD risk	Weigh the benefits and risks; consider if there is no history of bleeding and are willing to take it for ten years	ACC and AHA Guidelines	Grade C recommendation
Statins	PLWH with history of vascular disease or high CVD risk	Mandatory regardless of cholesterol levels; consider PCSK9 inhibitors and/or bempedoic acid if high-risk	European AIDS Clinical Society guidelines [109]	Reduces LDL-C, beneficial
	PLWH on protease inhibitors/ritonavir	Avoid simvastatin; use atorvastatin and rosuvastatin at low doses	Clinical recommendations	Avoid due to drug–drug interactions
Ezetimibe	PLWH not meeting LDL-C treatment targets	Considered if statin is not well tolerated or LDL-C targets are not met	Clinical trial (Saeedi et al.) [115]	Comparable LDL-C reduction, well-tolerated
PCSK9 inhibitors (Evolocumab)	PLWH on maximally tolerated statin therapy	Reduced LDL-C significantly over 24 weeks, safe and well-tolerated	Double-blind trial (Boccara et al.) [116]	Significant LDL-C reduction, safe

## 8. Conclusions

The relationship between HIV infection and cardiovascular health is complex, involving patients’ risk factors and habits, the direct and indirect impacts of HIV, and the effects of ART. HIV proteins (e.g., Tat, Nef, and gp120) contribute to atherosclerosis by promoting endothelial dysfunction and inflammation. Chronic inflammation and immune activation persist even with effective viral suppression, elevating cardiovascular risks. Metabolic impairments, such as dyslipidemia and insulin resistance, are prevalent in PLWH and are exacerbated by certain ART regimens. Statins and PCSK9 inhibitors show promise in managing dyslipidemia and reducing cardiovascular events in PLWH.

However, significant gaps remain in our understanding of the precise mechanisms by which HIV infection exacerbates cardiovascular diseases. For instance, the long-term effects of chronic immune activation on the cardiovascular system are not fully understood, nor is the exact role of specific HIV proteins in accelerating atherosclerosis. Additionally, the interplay between ART-induced metabolic changes and cardiovascular risk requires further exploration, particularly in understanding how different ART regimens influence lipid profiles, insulin resistance, and vascular inflammation over time.

Future research should focus on elucidating these mechanisms, including the role of immune activation and chronic inflammation in the development of cardiovascular diseases in PLWH. Longitudinal studies are needed to assess the long-term cardiovascular outcomes of different ART regimens, considering not only their efficacy in viral suppression but also their metabolic and cardiovascular side effects. Moreover, research should investigate the potential for novel therapeutic approaches that target the specific inflammatory pathways activated by HIV, with the goal of reducing cardiovascular risk without compromising viral control.

A comprehensive, multidisciplinary approach is essential for optimizing cardiovascular care in PLWH, balancing the benefits of ART with the need to minimize cardiovascular risks. To address these issues and improve the quality of life for PLWH, ART regimens could be optimized by tailoring them to individual patient profiles, including genetic factors, comorbidities, and potential drug–drug interactions, to minimize metabolic and cardiovascular side effects. Investment in the development of newer ART drugs that have fewer cardiovascular side effects while maintaining viral suppression efficacy is also crucial.

Comprehensive cardiovascular risk management should involve routine assessments, including lipid profiles, blood pressure, and markers of inflammation, for early detection and management of cardiovascular diseases. Prevention measures for high-risk patients, such as the use of statins, PCSK9 inhibitors, and antihypertensive medications, should be encouraged. Lifestyle interventions, including a heart-healthy diet and regular physical activity, are key to managing weight, improving lipid profiles, and reducing inflammation. Additionally, resources and support for smoking cessation are essential, as smoking significantly increases cardiovascular risk in PLWH.

Clinicians should address chronic inflammation by exploring the use of anti-inflammatory drugs that have shown promise in reducing cardiovascular events in the general population. Improving gut health by restoring gut microbiota balance through probiotics, prebiotics, and dietary modifications could also reduce systemic inflammation and improve overall health.

Finally, forming multidisciplinary care teams that include infectious disease specialists, cardiologists, dietitians, and mental health professionals is crucial for providing comprehensive care tailored to the needs of PLWH. Addressing mental health issues, such as depression and anxiety, through counseling and psychiatric care is important, as these conditions can negatively impact cardiovascular health and adherence to ART. Enhancing patient education on the importance of cardiovascular health, lifestyle modifications, and adherence to treatment regimens through workshops, informational materials, and personalized counseling is also essential.

## Data Availability

Not applicable.
